# Functional differentiation of stem cell-derived neurons from different murine backgrounds

**DOI:** 10.3389/fncel.2014.00049

**Published:** 2014-02-20

**Authors:** Lydia Barth, Rosmarie Sütterlin, Markus Nenniger, Kaspar E. Vogt

**Affiliations:** Department of Neurobiology/Pharmacology, Biozentrum, University of BaselBasel, Switzerland

**Keywords:** embryonic stem cells, neurons, electrophysiology, development, synaptic transmission

## Abstract

Murine stem cell-derived neurons have been used to study a wide variety of neuropsychiatric diseases with a hereditary component, ranging from autism to Alzheimer’s. While a significant amount of data on their molecular biology has been generated, there is little data on the physiology of these cultures. Different mouse strains show clear differences in behavioral and other neurobiologically relevant readouts. We have studied the physiology of early differentiation and network formation in neuronal cultures derived from three different mouse embryonic stem cell lines. We have found largely overlapping patterns with some significant differences in the timing of the functional milestones. Neurons from R1 showed the fastest development of intrinsic excitability, while E14Tg2a and J1 were slower. This was also reflected in an earlier appearance of synaptic activity in R1 cultures, while E14Tg2a and J1 were delayed by up to 2 days. In conclusion, stem cells from all backgrounds could be successfully differentiated into functioning neural networks with similar developmental patterns. Differences in the timing of specific milestones, suggest that control cell lines and time-points should be carefully chosen when investigating genetic alterations that lead to subtle deficits in neuronal function.

## Introduction

For a variety of neuropsychiatric disorders, disturbed neuronal development represents one of the most compelling hypotheses for their aetiology. In several cases, relatively mild defects in neuronal and synaptic signaling were found to be likely to cause the disease. For the vertebrate central nervous system (CNS) early developmental stages are difficult to investigate functionally, since the tissue is difficult to assess. Murine embryonic stem (mES) cell-derived neurons provide substantial methodological benefits for the study of functional aspects of such neuropsychiatric disorders, particularly those with a hereditary component. Essentially unlimited numbers of identical cultures can be produced and the cultures provide ready access to a developing neural system. These cultures have provided useful insights into the early stages of neuronal differentiation (Bibel et al., 2007) and have allowed studying a wide range of diseases from Rett Syndrome to Parkinson’s disease. Most of the investigators have used morphological, molecular and histochemical markers to describe their cultures. For several, especially neurodevelopmental disorders, such as autism, subtle networking deficits have been described (Ebert and Greenberg, 2013) and are hypothesized to cause the disease. Physiological data on the development of mES cell-derived neurons and neuronal networks are relatively scarce. We have therefore decided to investigate the physiology of developing neural networks formed by neurons derived from mES cells. Because different strains of mice have shown different neuropsychiatric phenotypes (Scharf and Schmidt, 2012; Barkus, 2013) we have decided to investigate cells from three different lines: (1) R1 (Nagy et al., 1993); (2) E14Tg2a (Stryke et al., 2003); and (3) J1 (Li et al., 1992). We investigated intrinsic neuronal parameters such as the resting membrane potential (RMP), the expression of voltage gated ion channels and the firing of action potentials (APs). We also studied the development of both excitatory and inhibitory synaptic contacts and their functional maturation. In addition we show morphological data on cultures derived from R1 cells, to provide a basis for comparison with existing data on neural differentiation from mES cell-derived neurons.

## Materials and methods

### Cell culture and differentiation

Three different lines of embryonic stem cells derived from wild type (wt) backgrounds R1, E14Tg2a and J1 were cultured and differentiated into neurons as described (Bibel et al., 2007). Briefly, after 4 days of embryoid body formation they are treated with 5 µM all-*trans*-retinoic acid (Sigma-Aldrich, Buchs, Switzerland) for additional 4 days. Embryoid bodies were dissociated and neuronal precursors were plated on poly-L-ornithine (Sigma)/laminin (Roche Inc., Buchs, Switzerland)—coated glass cover slips (Assistent, Karl Hecht GmbH, Sondheim/Rhön, Germany). At day *in vitro* (DIV) 0 and 1 neuronal precursors were cultured in neural medium containing DMEM/F12, N-2 Supplement (100X) and penicillin/streptomycin and 1 mM glutamine (all Invitrogen Inc., Lucerne, Switzerland). From DIV 2 the medium was changed to the differentiation medium containing Neurobasal® medium, B-27® Supplement (50X), N-2 Supplement (100X), 0.6 mM glutamine and penicillin/streptomycin (all Invitrogen).

### Electrophysiology

Cover slips with neurons were transferred to a bath chamber mounted to an inverse microscope (Axiovert 25, Carl Zeiss GmbH, München, Germany). Experiments were performed on DIV 0–8 and DIV 11–23 neurons in culture using the whole-cell voltage-clamp technique. Data were obtained using a Multiclamp 700A amplifier (Axon Instruments, Sunnyvale, CA). We used electrodes with an open tip resistance of 4–5 MΩ obtained by pulling borosilicate pipettes (Clark, Warner Instruments Inc., Edenbridge, UK) with 1.5 mm external diameter and 1.17 mm internal diameter without filament to a tip diameter of 1 µm on a horizontal Puller (DMZ Puller, Zeitz GmbH, Martinsried, Germany). The intracellular solution was adapted to the culture medium the cells were cultivated in; for N2 medium it contained (mM): 110 K-D-gluconate, 5 KCl, 11 Tris-phosphocreatine, 1 EGTA, 4.5 MgATP, 10 HEPES, 0.3 Tris-GTP (pH 7.4 with KOH, 290 mOsm). The extracellular solution for cells coming from N2 medium used for DIV 0 and DIV 1 contained (in mM): 120 NaCl, 29 NaHCO_3_, 4 KCl, 1 CaCl_2_, 0.7 MgCl_2_, 18 glucose, pH 7.4 when bubbled continuously with 95% O_2_ and 5% CO_2_. Intracellular solution for cultures coming from complete medium contained (mM): 100 K-D-gluconate, 5 NaCl, 1 EGTA, 5 MgATP, 10 HEPES, and 0.5 Tris-GTP (pH 7.4 with KOH, 210 mOsm). The extracellular solution for complete medium contained (in mM): 125 NaCl, 26 NaHCO_3_, 1.25 NaH_2_PO_4_*H_2_O, 2.5 KCl, 1.0 MgSO_4_, 2.0 CaCl_2_ and 11 glucose, pH 7.4 when bubbled continuously with 95% O_2_ and 5% CO_2_. Voltage-gated sodium- and potassium channels were detected in voltage-clamp mode at a holding potential of −60 mV. The holding potential was changed in a stepwise fashion from −75 mV to +25 mV in 5 mV increments and the voltage-gated peak inward current and maximal sustained outward current were measured. The inward currents were tetrodotoxin (TTX) sensitive, while the outward currents were blocked by tetraethyl-ammonium (TEA) (3 mM) and 4-aminopyridine (4-AP) (1 mM). RMP and APs were recorded in current-clamp mode. Somatic current injections were applied in 2.5 pA steps from −2.5 pA to +30 pA. Synaptic activity was measured in voltage-clamp mode: to detect spontaneous excitatory postsynaptic currents (sEPSCs), cells were held at −60 mV, while for spontaneous inhibitory postsynaptic currents (sIPSCs), cells were held at −40 mV. Responses were filtered at 5 kHz and digitized at 20 kHz. The excitatory glutamate receptor blocker 2,3-dihydroxy-6-nitro-7-sulfamoyl-benzo[f]quinoxaline-2,3-dione (NBQX) (10 µM) and antagonists of inhibitory GABA_A_ receptors picrotoxin (100**** µM) or bicuculline (20 µM) were added to the perfusate to block the respective synaptic activity (Tocris Inc., Bristol, UK). Recorded sEPSC and sIPSC were detected and analysed using Mini Analysis 6 (Synaptosoft, Decatur, GA). All other data analysis was done with IGOR PRO 6.0 (Wavemetrics, Lake Oswego, OR) software.

### Immunocytochemistry

Cells cultured on glass coverslips were rinsed twice with phosphate buffered saline (PBS) pH 7.4 and fixed with 10% neutral buffered formalin (Sigma-Aldrich) for 20 min at room temperature (RT). After rinsing with PBS, coverslips were permeabilized for 5 min in 0.2% TritonX-100/PBS, rinsed with PBS and incubated for 1 h at RT in a humidified chamber with the following primary antibodies and dilutions (rb: rabbit, ms mouse): doublecortin (rb, 1:1000, Cell Signaling, Bio Concept, Allschwil Switzerland), microtubule-associated protein 2 (MAP2) (rb, 1:1000, Chemicon, Millipore Inc., Zug, Switzerland) and synaptophysin (ms, 1:300, Sigma Aldrich). After several washes with PBS, coverslips were incubated for 1 h at RT with corresponding secondary antibody: Cy3 (goat anti rabbit IgG (H+L) 1:1000)****, Cy5 (goat anti rabbit IgG (H+L), 1:200), (Immuno Jackson, Suffolk UK), Alexa 488-phalloidin, 1:400, (Molecular Probes, Eugene, OR) and 4’, 6-diamidino-2-phenylindole (DAPI) (1:1000, Molecular Probes). After several washes in PBS, coverslips were mounted in Mowiol-1188 as previously described (Baschong et al., 1999). Confocal sections were recorded with a confocal laser scanning microscope Leica time confocal scanner (TCS) SPE with DMI 4000B (Leica Switzerland) and processed with Imaris software (Bitplane, Zurich, Switzerland) and Adobe Photoshop version 10.0 (Adobe Inc., California).

### Statistics

Results are given as mean ± standard error of the mean (SEM). Statistical analysis usually involved comparisons between cells from all three backgrounds. After testing for normality we therefore used two-way analysis of variance (ANOVA) tests, followed by *post-hoc* Bonferroni tests, where appropriate. Values below 0.05 were considered significant.

## Results

### Morphological development

**Figure 1:**
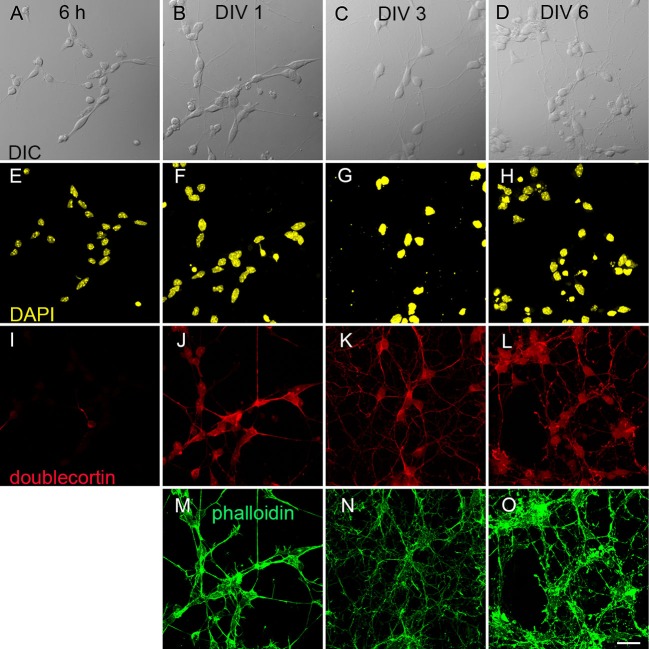
**Derivation of neurons from mouse embryonic stem cells. (A–D)** differential interference contrast (DIC) images of early neuronal differentiation of R1 stem cell-derived cultures. **(A)** Progenitor cells 6 h after plating; **(B)** at DIV 1; **(C)** at DIV 3 and **(D)** at DIV 6. **(E–O)** Immunostainings against neuronal markers at the corresponding DIV. **(E–H)** Nuclear staining with DAPI. **(I–L)** Labeling against the marker for immature neurons doublecortin. **(M–O)** Actin staining with phalloidin. Scale bar: 20 µm.

We differentiated R1, E14Tg2a and J1 stem cells into neurons using an established protocol for mES cells (Bibel et al., 2007). Progenitor cells showed already on DIV 0 a clearly spindle shaped morphology (Figure 1A). Over the course of further development all cells acquired a distinct multipolar shape (Figure 1B–D). We followed the morphological development from DIV 0 to DIV 6 using R1 cells as an example. Cells were immunostained with antibodies against the nuclear marker DAPI (Figure 1E–H), the neuronal marker doublecortin (Figure 1I–L) and stained with the actin cytoskeleton label phalloidin (Figure 1M–O). These stainings reveal that on DIV 1 85% of cells expressed doublecortin and this fraction rose to >90% by DIV 6. This developmental pattern was found in all stained cultures.

### Functional development

**Figure 2:**
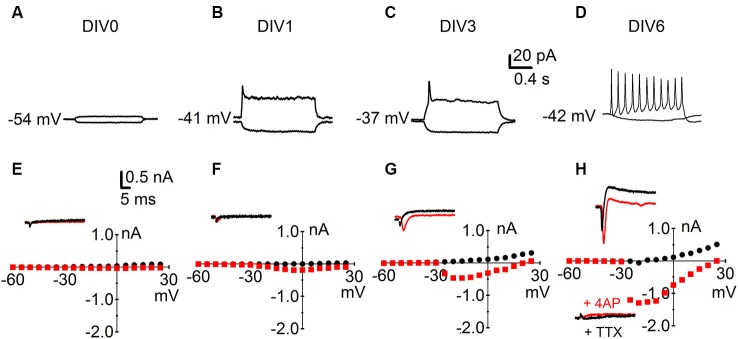
**Characterization of intrinsic parameters in immature developing neurons.** Early development of neuronal physiology; the numbers next to the traces indicate the RMP. **(A–D)** Reaction of R1 neurons to negative (bottom traces) and positive (top traces) somatic current injections at **(A)** DIV 0; **(B)** DIV 1; **(C)** DIV 3 and **(D)** DIV 6. **(E–H)** Sample traces and I-V curves for voltage-activated inward sodium currents (red) and voltage-activated outward potassium currents (black). Data points for the I-V curves plot peak inward current (red squares) against the holding potential after depolarization and the sustained outward current (black dots) against the holding potential after depolarization. Corresponding time points: **(E)** DIV 0; **(F)** DIV 1; **(G)** DIV 3 and **(H)** DIV 6 top insert currents under baseline condition and bottom insert currents after bath application of TTX (1 µM) and 4 AP (1 mM).

We next investigated key physiological parameters of neuronal maturation. We obtained patch-clamp recordings from cells throughout their neuronal differentiation from DIV 0 to DIV 13. Cells were held in the current-clamp configuration and their RMP was recorded; somatic current injection was then used to investigate AP firing in the developing neurons (Figure 2A–D). Cells usually started firing immature single APs at DIV 3; by DIV 6 most neurons produced repetitive, mature APs upon a 0.8 s depolarizing pulse. The same cells were subsequently held in the voltage-clamp configuration and the functional expression of voltage-gated conductances was studied (Figure 2E–H). Depolarizing voltage steps produced two types of currents: (1) fast activating and inactivating inward currents; and (2) slow activating outward currents with little inactivation. Inward currents were blocked by the sodium channel blocker TTX (1 µM), while outward currents were sensitive to the potassium channel blockers TEA (3 mM) and 4-AP (1 mM) (*n* = 15) (Figure 2H).

**Figure 3:**
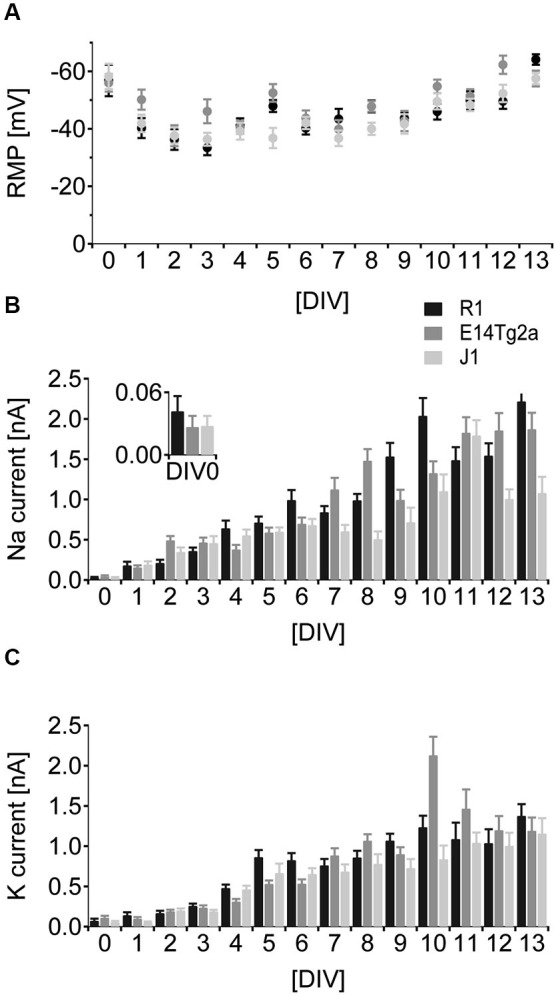
**Developmental characterization of RMP and voltage gated sodium- and potassium currents. (A)** Comparison of RMP from DIV 0-13 of neurons derived from R1 (black), E14T2ga (gray) and J1 (light gray) mES cells. **(B)** Comparison of inward sodium currents as a function of developmental age from DIV 0-13 in neurons derived from R1 (black), E14T2ga (gray) and J1 (light gray) mES cells. **(C)** Comparison of outward potassium currents as a function of developmental age from DIV 0–13 in neurons derived from R1 (black), E14T2ga (gray) and J1 (light gray) mES cells.

In cells from all backgrounds RMP development followed a characteristic time-course. After an initial hyperpolarized phase at DIV 0 cells depolarized over the following days and started hyperpolarizing again from DIV 5 (Figure 3A). By DIV 13 neurons in culture showed a mature, hyperpolarized RMP of −64 mV ± 1.8 mV for R1, −58 mV ± 2.7 mV for E14Tg2a and −57 mV ± 2.0 mV for J1 cultures. Statistical analysis revealed a highly significant influence (*p* < 0.01) of developmental age and of the cell type on RMP, but no interaction between the two variables (*n* > 15 measurements per day and background; two-way ANOVA). *Post-hoc* comparisons (Bonferroni test) showed that changes in RMP were largest for early time-points. Significant differences between cell lines were restricted to DIV 0.

Voltage-dependent sodium currents show a monotonous increase in amplitude from DIV 0 to DIV 13 in cells from all backgrounds (Figure 3B). We found a highly significant (*p* < 0.01) influence of DIV and background on the amplitude of voltage-gated sodium currents—the interaction between the two variables was also highly significant (*p* < 0.01) (*n* = 25 per cell type and day; two-way ANOVA). The difference between backgrounds was significant for DIV 0 (*post-hoc* Bonferroni test). In cells from all backgrounds, voltage-dependent potassium currents showed an early increase that leveled at around 1.5 nA at DIV 8 (Figure 3C). Again the current amplitude was highly significantly dependent on DIV and background with a significant interaction between the two (*p* < 0.01 for all, *n* = 25 per cell type and day, two-way ANOVA). In *post-hoc* comparisons the influence of the background was restricted to DIV 0 (*p* < 0.05, Bonferroni test).

### Action potentials and excitability

**Figure 4:**
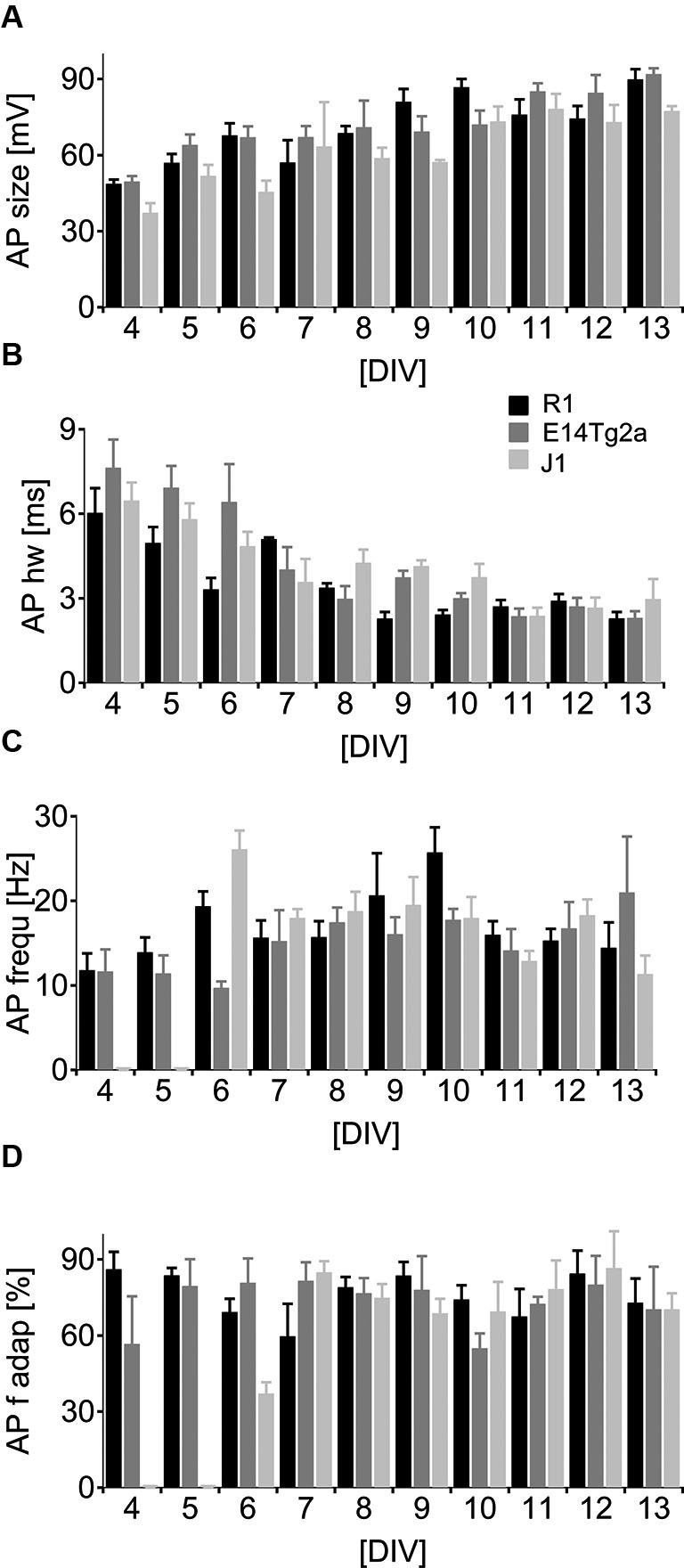
**Neuronal spiking patterns as a function of developmental age. (A–D)** Analysis of the spiking parameters in neurons derived from of R1 (black), E14T2ga (gray) and J1 (light gray) mES cells as a function of time from DIV 0–13 in culture. Cells were subject to a 0.8 s depolarizing somatic current pulse. **(A)** Data for the amplitude of the first AP, **(B)** the half-width at half height for the first AP, **(C)** the initial firing frequency and **(D)** the frequency at the end of the train relative to the initial firing frequency.

To study AP generation by the developing neurons, we depolarized cells above threshold by somatic current injection in current-clamp mode. In very young cells, either no active depolarization or only an immature spike was detected (see Figure 2B, C). As the cultures matured the APs grew significantly in size (Figure 4A) (*p* < 0.01) and became significantly faster (Figure 4B) (*p* < 0.01, *n* = 103 for both parameters, two-way ANOVA). By DIV 6, most cells fired repetitive, fast APs (see Figure 2D) during a 0.8 s long depolarizing pulse. There was no significant dependence of the peak AP frequency (Figure 4C) (*p* > 0.40, *n* = 95, two-way ANOVA) or of the amount of frequency adaptation (Figure 4D) on DIV (*p* > 0.93, *n* = 89, two-way ANOVA). When comparing cells from different backgrounds we found a highly significant influence of the cell type on AP half-width and on the peak AP frequency (*p* < 0.01; *n* = 94 for each cell type, two-way ANOVA).

### Synaptic activity

**Figure 5:**
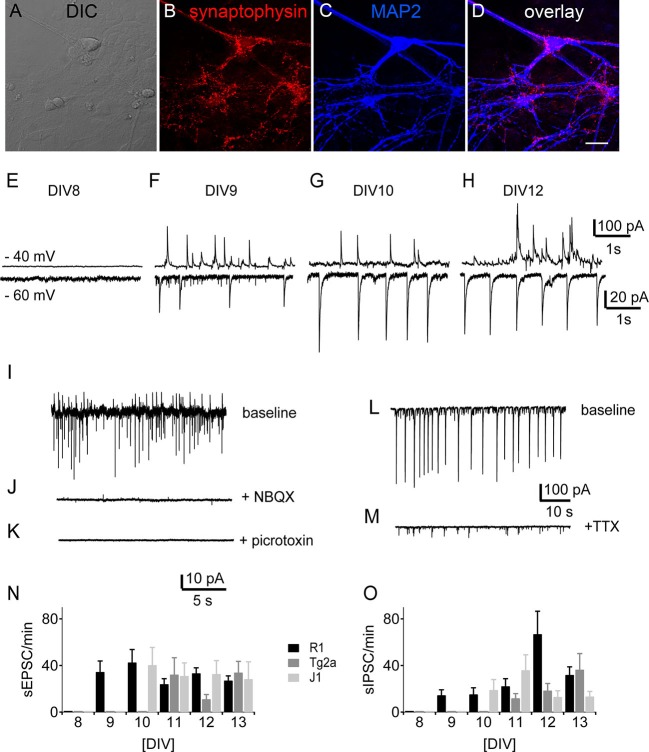
**Development of synaptic activity.** Morphology of synaptic contacts: DIC **(A)** and immunostainings **(B–D)** of DIV 12 R1 cells against the presynaptic marker synaptophysin **(B)**, the neuronal marker MAP2 **(C)** and overlay of both **(D)**. Scale bar: 20 µm. **(E–H)** Traces of spontaneous synaptic activity of R1 cells in voltage-clamp from DIV 8, DIV 9, DIV 10 and DIV 12. Top traces at −40 mV holding potential, bottom traces at −60 mV respectively. **(I–K)** Pharmacological characterization of synaptic currents. **(I)** Sample trace of one experiment with baseline recording **(J)** after bath application of NBQX (10 µM) and **(K)** bath application of picrotoxin (100 µM). **(L)** Sample trace of spontaneous synaptic activity under control conditions and **(M)** after the application of TTX (1 µM). **(N)** Frequency of sEPSCs as a function of DIV for neurons derived from R1 (black), T2ga (gray) and J1 (light gray) mES cells in cultures from DIV 8–13. **(O)** Same data for sIPSCs.

Punctate labeling for the synaptic marker synaptophysin could be observed in close contact to the postsynaptic label MAP2 (Figure 5A–D) in R1 mES cell-derived cultures at DIV 12. Such stainings were observed in all cultures tested (data not shown), suggesting that synapses had formed by then. We therefore looked for spontaneous synaptic events in voltage-clamp recordings from cell beginning with DIV 8. At DIV 8 none of the recorded cells showed any synaptic activity (*n* = 10 for the different backgrounds). We detected the first spontaneous excitatory (sEPSCs) and inhibitory (sIPSCs) postsynaptic currents by DIV 9 in R1 (*n* = 15) neurons, by DIV 10 in J1 (*n* = 6) and by DIV 11 in E14Tg2a (*n* = 15) neuronal cultures (Figure 5). We measured sEPSCs as inward currents (Figure 5E–H, bottom traces) at a holding potential of −60 mV, while sIPSCs were best visible and measured as outward currents at a holding potential of −40 mV (Figure 5E–H, top traces). The sEPSCs were blocked by the addition of the AMPA-receptor blocker NBQX (10 µM) to the perfusate (Figure 5J), whereas sIPSCs were blocked by bath application of the GABA_A_ receptor antagonist picrotoxin (100 µM) (Figure 5K) in all cultures tested (*n* = 10). Spontaneous synaptic activity was sensitive to the application of the sodium channel blocker TTX (1 µM) (*n* = 13) (Figure 5L, M). Apart from the different ages of onset there was no significant difference in the number of either sEPSCs or sIPSCs between cells from different backgrounds (Figure 5N, O) (*n* = 178, two-way ANOVA).

## Discussion

Here we show that mES cell-derived neurons mature within >2 weeks in culture and form functional, synaptically connected networks around DIV 11. The morphological data we collected on the R1 line follows published data (Bibel et al., 2007). Neurons undergo a relatively stereotypical development; thus, for the lines tested here the exact background of the mES cells is not a decisive factor when designing such experiments. We therefore refrained from an extensive morphological analysis of all three lines. For all of the parameters measured, we observed a considerable variability from cell to cell and from culture to culture, necessitating a large number of measurements to determine a given data point. The RMP shows an interesting biphasic behavior: from an initially hyperpolarized state cells undergo transient depolarization and then gradually repolarize to values that are typical for mature neurons. While the overall behavior is the same for cells derived from different mES backgrounds, there are significant differences in the time-course for the different lines.

Rudimentary APs were detected surprisingly early during development, however mature overshooting spikes were reliably evoked only in cultures beginning at DIV 6. The maturation in AP-shape was paralleled by the increasing function of voltage-gated conductances in these cultures. Both voltage-gated sodium-, as well as potassium conductances showed a several fold increase in amplitude over the time studied. Again, cells from different backgrounds showed the same general pattern with significant differences in timing early on. Excitatory and inhibitory synaptic events were detected at the same time for cultures from the same background. This argues against an accelerated maturation of one type of synapse, as has been described for several *in vivo* situations (Ben-Ari, 2002). APs contribute to synaptic activity in these cultures, as the effect of TTX on spontaneous synaptic events shows. The occurrence of synaptic activity did not parallel the different speed at which voltage-gated conductances developed. Cultures from J1 background were delayed in their development of voltage-gated conductances, but showed synaptic activity early on, while cells from E14Tg2a background developed their voltage-gated conductances relatively fast, but were delayed in showing synaptic activity compared to cells from other backgrounds. This indicates that the differences between cultures from different backgrounds are not due to a “master-clock” running at different speeds, but rather a subtly different developmental pattern in the different mES strains. While cell density might influence the onset of synaptic activity, it is less obvious how it would affect the maturation of intrinsic parameters. We carefully controlled the plating density of the cells and can rule out a systematic difference between the different cell lines at DIV 0. Cell density in mature cultures was not obviously different, but subtle differences might have contributed some of the differences in the occurrence of synaptic events. While the differences in developmental timing were relatively small, they can become significant and this may affect the comparison between wt and genetically modified neurons. Compared to differentiating murine neurons in situ, measured in timed acute brain slice preparations, the mES derived-neurons showed similar developmental milestones. For voltage-gated sodium- and potassium currents similar mature values were reached (I_Na_ 1.7 nA in mES vs. 0.8 nA in situ; I_K_ 1.3 nA in mES and 1.2 nA in situ) (Picken Bahrey and Moody, 2003). The resulting APs were also very similar in the development of their kinetics. In situ neurons took 19 days (E14 to P12) to mature, while mES cell-derived neurons took 16 days (4 days of retinoic acid treatment followed by plating and 12 days of maturation) to reach comparable developmental stages (Picken Bahrey and Moody, 2003). This indicates that the development of the intrinsic neuronal physiology is surprisingly faithfully reproduced by mES cell-derived neurons. In comparison to neurons derived from neural stem cells (NS) expanded after differentiation from mES (Biella et al., 2007) our cells, which do not undergo further expansion, develop more rapidly. They produce repetitive fast APs at DIV 6, while the NS derived neurons reach that milestone not before DIV 10 or possibly later.

## Conclusion

Neurons differentiated from mES cells show a robust developmental pattern across several strains of ES cells and form mature neural networks within a time frame comparable to the *in vivo* situation. This makes mES cell-derived neuronal cultures a straightforwardly accessible system that can be easily treated with different pharmacological compounds or modulators of gene expression.

## Author contributions

Lydia Barth: conception and design, data collection, analysis and interpretation, manuscript preparation. Rosmarie Sütterlin: conception and design, data collection, analysis and interpretation. Markus Nenniger: data analysis and interpretation. Kaspar Emanuel Vogt: conception and design, data analysis and interpretation, manuscript preparation.

## Conflict of interest statement

The authors declare that the research was conducted in the absence of any commercial or financial relationships that could be construed as a potential conflict of interest.
